# Endoscopic Management of Eosinophilic Esophagitis: A Narrative Review on Diagnosis and Treatment

**DOI:** 10.3390/jcm14113756

**Published:** 2025-05-27

**Authors:** Andrea Pasta, Francesco Calabrese, Manuele Furnari, Edoardo Vincenzo Savarino, Pierfrancesco Visaggi, Giorgia Bodini, Elena Formisano, Patrizia Zentilin, Edoardo Giovanni Giannini, Elisa Marabotto

**Affiliations:** 1 Gastroenterology Unit, Department of Internal Medicine, University of Genoa, 16132 Genoa, Italy; calabrese.francesco.93@gmail.com (F.C.); manuele.furnari@unige.it (M.F.); giorgia.bodini@unige.it (G.B.); pzentilin@unige.it (P.Z.); elisa.marabotto@unige.it (E.M.); 2 IRCCS Policlinic San Martino Hospital, 16132 Genoa, Italy; 3 Department of Surgery, Oncology and Gastroenterology, University of Padua, 35128 Padua, Italy; 4 Gastroenterology Unit, University Hospital of Padua, 35128 Padua, Italy; 5 Division of Gastroenterology, Department of Translational Research and New Technologies in Medicine and Surgery, University of Pisa, 56126 Pisa, Italy; pierfrancesco.visaggi@gmail.com

**Keywords:** eosinophilic esophagitis, endoscopy, biopsy, esophageal dilation, EndoFlip™, EREFS, strictures, disease monitoring

## Abstract

Eosinophilic esophagitis (EoE) is a chronic, immune-mediated disease characterized by esophageal eosinophilic infiltration, leading to symptoms such as dysphagia and food impaction. Endoscopy is central to both diagnosis and management, allowing for the direct visualization of characteristic features, biopsy collection, and therapeutic interventions. Despite its diagnostic value, up to one-third of patients may present with a normal-appearing esophagus, highlighting the importance of standardized scoring systems and a systematic biopsy approach. This review explores the evolving role of endoscopy in EoE, from traditional diagnostic methods to emerging technologies such as EndoFlip™ for assessing esophageal distensibility, transnasal endoscopy for non-sedated monitoring, and novel dilation techniques for fibrostenotic disease. Additionally, non-invasive biomarkers and minimally invasive tools are reshaping disease monitoring. By integrating endoscopic, histologic, and molecular approaches, future advancements may enhance diagnostic accuracy and optimize personalized treatment strategies for EoE.

## 1. Introduction

Eosinophilic esophagitis (EoE) is a chronic, immune-mediated inflammatory disease of the esophagus characterized by a high density of eosinophils in the esophageal tissue [1,2]. EoE has become increasingly recognized in recent decades as a distinct disease, differing from other esophageal conditions causing esophageal eosinophilia, such as gastroesophageal reflux disease (GERD), due to its specific immunological profile and response to allergens [3,4].

The pathophysiology of EoE involves a complex interplay of genetic and environmental factors, resulting in an immune response dominated by T-helper type 2 (Th2) pathway [5]. In individuals with genetic predisposition, exposure to dietary antigens or environmental triggers leads to the release of cytokines, such as IL-33, IL-25, and thymic stromal lymphopoietin (TSLP), which promote a Th2 response [6]. This cascade activates cytokines like IL-13, IL-4, and IL-5, which, in turn, recruit eosinophils, mast cells, and other inflammatory cells to the esophageal mucosa [7,8]. Over time, chronic inflammation can disrupt the esophageal barrier, leading to tissue remodeling, fibrosis, and stricture formation [9].

Epidemiologically, the incidence and prevalence of EoE have increased significantly in recent years, with current estimates indicating an incidence of approximately 20 cases per 100,000 per year and a prevalence of about 1 case per 1000; more recent estimates indicate a current prevalence of greater than 150 cases per 100,000 in the US. This rise is partly due to increased disease awareness and improved diagnostic practices, though evidence suggests a true increase in EoE incidence [10,11,12]. The condition is more common in males, individuals with a history of atopic diseases, and those with a family history of EoE, pointing to both environmental and genetic contributions to disease susceptibility [13].

The clinical impact of EoE is significant, as it affects patients’ quality of life and can lead to serious complications particularly if untreated [14,15]. In adults, the primary symptoms include dysphagia, especially with solid foods, and food impaction, which may sometimes require emergency endoscopic intervention to clear the esophagus [16,17]. Food bolus impaction is a common and recurring issue in EoE, often necessitating dietary and behavioral adaptations [18]. Patients frequently adopt practices such as chewing food excessively, eating slowly, and drinking water to facilitate swallowing, which can mask symptoms and delay diagnosis [19]. This often leads to a prolonged period before diagnosis, with a median overall diagnostic delay of 36 months (IQR 12–88), and patient-dependent and physician-dependent delays are 18 months (IQR 5–49) and 6 months (IQR 1–24), respectively [20]. In this regard, it has been shown that adaptive behaviors modify the perception of dysphagia by patients with EoE, possibly masking symptoms [21]. EoE can also mimic GERD, with symptoms such as heartburn and regurgitation, further complicating diagnosis [22]. Exercise-induced chest pain is another symptom reported in adults [21,22,23].

In younger patients, the clinical presentation varies. Infants and young children tend to show nonspecific symptoms like feeding difficulties, vomiting, and failure to thrive, whereas adolescents and young adults more commonly experience symptoms related to esophageal fibrosis, such as dysphagia and food impaction [24]. Over time, untreated EoE can lead to progressive esophageal narrowing due to fibrosis, increasing the risk of strictures [1]. Studies indicate that the risk of esophageal stricture grows by about 9% with each year of untreated symptoms, highlighting the importance of early diagnosis and intervention [25].

Given the potential for severe complications and the chronic nature of EoE, early recognition and management are critical in reducing the disease’s impact on daily life and preventing long-term esophageal damage that could require endoscopic treatment.

Endoscopy plays a central role in diagnosing EoE by providing a direct visual assessment of the esophagus, allowing for biopsy sampling, and helping differentiate EoE from other causes of esophageal eosinophilia [26,27]. EoE can often present with subtle endoscopic signs, which makes careful examination and adherence to biopsy protocols critical for accurate diagnosis [27]. Endoscopy is crucial in assessing the presence and extent of characteristic EoE features [26], although these features may vary in severity, and up to 7–32% of patients can have a normal-appearing esophagus on endoscopy, particularly in the early stages of the disease [28]. As a result, endoscopists should maintain a high level of suspicion for EoE, especially in atopic patients presenting with symptoms such as dysphagia or food impaction [26]. To optimize diagnostic accuracy, endoscopists should be proficient in the assessment of the esophageal mucosa [29] and adhere closely to established guidelines for biopsy sampling [30,31,32].

## 2. Literature Gap, Aim, and Methodology

As we see it in our clinical practice, EoE is no longer a rarity but a condition encountered with increasing frequency, mirroring the documented rise in its incidence and prevalence worldwide [33]. In parallel, the resources for diagnosis and treatment have broadened—endoscopic distensibility measurements, less-invasive sampling devices, and targeted therapies now complement traditional approaches. Existing reviews tend to examine these advances one at a time, leaving clinicians without a unified perspective.

The aim of this article is to offer a comprehensive narrative review that synthesizes the latest evidence in endoscopy across diagnostic, therapeutic, and monitoring domains, and thus, provides practitioners and trainees with a practical “one-stop” guide for everyday decision-making and for identifying priority areas for future research.

To achieve this integrative objective, we adopted a narrative review methodology. We searched PubMed, Embase, and Scopus, pairing “eosinophilic esophagitis” with endoscopy-focused keywords (“endoscopic”, “dilation”, “impedance”, “planimetry”, “capsule”, “biopsy”, “surveillance”, “monitoring”, “therapy”). Two reviewers independently screened titles, abstracts, and full texts, retaining human studies deemed seminal, methodologically sound, or reflective of emerging trends. We considered meta-analyses, narrative and systematic reviews, consensus statements, and guidelines that addressed any endoscopic or minimally invasive aspect of EoE management.

## 3. Endoscopic Diagnosis of Eosinophilic Esophagitis

### 3.1. Endoscopic Findings

Endoscopic examination is an essential component in diagnosing EoE, as it allows clinicians to directly visualize characteristic esophageal changes and collect biopsy samples for histopathological analysis [30]. EoE typically presents with distinctive endoscopic findings, although these may vary across patients and disease stages [27,34].

Common endoscopic findings in EoE include:

⮚ Major findings
  - Mucosal edema: The esophagus may show pallor or loss of vascular markings, indicative of inflammation and mucosal swelling [35].
  - Esophageal rings or “trachealization”: This feature, marked by concentric rings in the esophagus, affords it a ridged appearance resembling the trachea [36]. Rings may be subtle or prominent, especially in long-standing disease, and can be a cause of dysphagia [37].
  - White exudates or plaques: These small, white spots or patches on the mucosal surface indicate clusters of eosinophils and may be confused with candida infection. Exudates are frequently observed in active EoE inflammation [35,38].
  - Linear furrows: Longitudinal grooves or furrows along the esophagus are common in EoE and are visible in the inflamed mucosa. These linear markings often extend throughout the length of the esophagus [35].
  - Strictures: Strictures, or narrowing of the esophageal lumen, can develop as a result of chronic inflammation and fibrosis in advanced EoE cases [15]. They contribute to the symptoms of dysphagia and food impaction that characterize EoE’s clinical presentation [34].
⮚ Minor findings
  - “Crepe-Paper” Esophagus: Fragile, easily tearable mucosa that may peel during endoscopy [39].
  - Granular or Nodular Mucosa: Granular appearance due to subtle irregularities on the mucosal surface [40].
  - Mucosal Tears or Rents: Tearing of the esophageal lining during endoscopy, sometimes occurring spontaneously due to mucosal fragility [39].

Notably, up to 7–32% of patients with EoE may have a normal-appearing esophagus on endoscopy, especially in early disease stages [28]. Therefore, an endoscopic examination is not sufficient for diagnosing EoE [41]. Moreover, the proportion of normal-appearing endoscopies can also depend on the endoscopist’s expertise, as subtle signs such as edema or other endoscopic findings may be overlooked [15,42].

In summary, endoscopic findings in EoE are varied and can sometimes be subtle, necessitating careful examination and a high index of suspicion [30]. When characteristic endoscopic features are present, they are highly indicative of EoE, but diagnosis should be confirmed with histologic analysis [43].

### 3.2. Endoscopic Severity Assessment: Endoscopic Activity Indexes

In recent years, several endoscopic scoring systems have been developed in the field of EoE to objectively quantify alterations and enhance disease monitoring in both clinical practice and research settings. One of the most widely used systems is the Eosinophilic Esophagitis Endoscopic Reference Score (EREFS), introduced by Hirano et al. [37]. This index grades key endoscopic features, including edema, rings, exudates, furrows, and strictures, with the aim of providing a standardized assessment of EoE severity [44]. Despite its utility, EREFS has limitations in fully capturing disease progression, particularly in distinguishing between inflammatory and fibrostenotic phenotypes and its reliability has been extensively evaluated [45]. Furthermore, the EREFS scoring system has shown limited capacity to track histological improvements, does not correlate with adaptive behaviors, and should not serve as the sole measure of disease activity [21].

In this context, Van Rhijn et al. found that individual endoscopic signs did not consistently predict peak eosinophil counts, and histologic remission often did not coincide with improved endoscopic findings, emphasizing the necessity of biopsies [46]. Ma et al. reported moderate-to-substantial inter-rater reliability for most EREFS components but noted significant variability in assessing strictures (ICC as low as 0.072), with no improvement when scoring the worst affected area [47].

To address some of these limitations, alternative and modified scoring systems have been proposed. Schoepfer et al. [48] developed regression-based models incorporating EREFS features, which showed good agreement with global endoscopic assessments (EndoGAs). These scoring models aimed to improve the consistency of endoscopic severity evaluation and enhance its predictive value in clinical trials [48]. Additionally, the new Index of Severity for Eosinophilic Esophagitis (I-SEE) was recently introduced as a comprehensive tool integrating endoscopic, histologic, and symptom-based parameters. This index classifies patients into mild, moderate, and severe categories based on a multidimensional assessment, acknowledging that endoscopy alone is insufficient to determine overall disease burden [49].

In conclusion, while endoscopic activity indices such as EREFS provide valuable insights into EoE severity, their limitations necessitate a multimodal approach. The integration of endoscopic findings with histologic and symptomatic data remains essential for accurate disease monitoring and treatment decision-making. Overall, ongoing debate remains regarding EREFS capacity to fully reflect clinical outcomes and distinguish between inflammatory and fibrostenotic phenotypes, highlighting a critical area requiring further research; we feel that additional studies integrating endoscopic findings with histological and patient-reported outcomes are essential to refine the EREFS scoring system, thereby improving its clinical relevance and ability to guide personalized treatment strategies.

### 3.3. Correct Biopsy Approach

Endoscopic appearance alone is not sufficient for EoE diagnosis, though biopsies should be performed regardless of visual findings in patients presenting with EoE symptoms, such as dysphagia or food impaction [31]. The accurate diagnosis of EoE requires a meticulous approach to esophageal biopsy. Due to the patchy nature of eosinophilic infiltration, where eosinophil density varies significantly within the esophagus, a well-structured biopsy protocol is crucial to capture diagnostic features [50,51,52]. Guidelines on biopsy quantity, location, and timing are essential to improve the diagnostic sensitivity for EoE [30].

Studies highlight the importance of collecting multiple biopsy samples to enhance diagnostic accuracy [43]. It is well-known that obtaining at least six biopsy specimens significantly increases diagnostic sensitivity, capturing a representative tissue sample even when eosinophils are unevenly distributed [53]. In one adult study, sensitivity reached 100% when five biopsy specimens were obtained, while a single specimen showed only 55% sensitivity [54]. Pediatric studies similarly show that diagnostic sensitivity improves progressively with the number of biopsies taken, achieving 100% sensitivity with six samples [55]. Based on these findings, the recommended approach is to obtain a minimum of six biopsies from different esophageal locations to capture the extent of eosinophilic infiltration [30,31,32].

In addition to the number, the location of biopsies is fundamental. Eosinophilic infiltration often differs along the length of the esophagus, with a greater density in the distal segment [50,52,56]. However, significant inflammation can also be present proximally; so, biopsies should ideally be collected from both the proximal and distal esophagus [43,57]. Wechsler et al. showed that, for diagnosing active EoE, distal biopsies have over 80% sensitivity, and reach a sensitivity of 65% and 62% at the middle and proximal esophagus, respectively [58]. Combining distal and proximal biopsies provides the highest sensitivity for two-site sampling [58]. Therefore, it is recommended that 2–4 biopsy samples be collected from the distal esophagus and 2–4 from the proximal esophagus, ideally targeting visibly inflamed areas [43,59]. The “turn and suction technique” should be the preferred choice for biopsy sampling since this allows the collection of larger and deeper biopsy samples [60]: The technique consists in advancing the biopsy forceps through the working channel of the endoscope, opening them and pulling them back toward the endoscope’s tip. The endoscope should then be tilted upward or downward at an angle of approximately 45 to 90 degrees and rotated toward the esophageal wall. Once the forceps are in contact with the esophageal wall, suction should be applied to draw the mucosal tissue inside the forceps. The forceps should then be closed to grasp the tissue and retracted to retrieve the biopsy sample [29].

For patients presenting with food impaction, a common initial symptom of EoE, biopsies should be collected during the emergency endoscopy [30,31]. It is recommended to avoid taking biopsies directly from the site of impaction, as trauma from impaction may cause acute changes in the mucosa that can obscure chronic inflammatory features [30]. Comprehensive biopsies during the initial endoscopic evaluation minimize the need for repeat procedures, facilitating a timely diagnosis and treatment initiation.

Additional biopsies from the stomach and duodenum may be considered in patients with symptoms suggestive of eosinophilic involvement beyond the esophagus, such as abdominal pain or nausea [30,61]. Although concurrent eosinophilic gastrointestinal disorders (EGIDs) are rare, their presence can inform treatment approaches [62]. This added layer of evaluation ensures that overlapping or coexisting conditions are addressed in the diagnosis and management plan.

### 3.4. Novel Adjunctive Diagnostic Tools

While traditional endoscopy and biopsy remain primary methods for diagnosing EoE, several novel adjunctive tools, including the esophageal string test, transnasal endoscopy, molecular biomarker analysis, immunohistochemistry, and Endoluminal Functional Lumen Imaging Probe (EndoFlip™ Medtronic, Crospon Inc., Minneapolis, MN USA) EndoFlip™, have expanded the diagnostic and monitoring capabilities for this condition [63].

One of the promising tools is EndoFlip™, a device used to assess the esophageal distensibility or flexibility by measuring the diameter and pressure within the esophageal lumen [64]. EndoFlip™ can help evaluate esophageal distensibility and can impact on dysphagia severity [65]. Hoffmann et al. showed in a pediatric population that decreased esophageal distensibility correlates with disease progression and fibrosis, making EndoFlip™ a valuable tool for monitoring EoE severity and potentially improving therapeutic decisions [66].

In addition to EndoFlip™, the esophageal string test (EST) is another adjunct that allows for the non-invasive monitoring of eosinophil activity by collecting esophageal secretions [67]. This tool, which measures eosinophil proteins like eosinophil-derived neurotoxin (EDN), has been shown to correlate well with biopsy findings, providing a useful option for follow-up assessments in patients with EoE [68].

Similarly, Cytosponge, a minimally invasive sampling device originally developed for Barrett’s esophagus, has shown promise in assessing histologic activity in EoE. Cytosponge consists of an ingestible gelatin capsule containing a compressed mesh sponge attached to a string. Once swallowed, the capsule dissolves in the stomach, allowing the sponge to expand and collect esophageal tissue upon retrieval through the mouth. A two-center study demonstrated that Cytosponge provided an accurate, safe, and well-tolerated method for assessing histologic activity in EoE, with high sensitivity (75%) and specificity (86%) compared to endoscopic biopsy. Importantly, patients in the study preferred Cytosponge to endoscopy, highlighting its potential as a less invasive monitoring tool in EoE management [69].

Additionally, transnasal endoscopy (TNE) provides a sedation-free, less invasive endoscopic option that can effectively monitor disease progression in EoE patients [70,71]. TNE is especially valuable for ongoing assessments in treatment monitoring, as it reduces patient discomfort and eliminates the need for anesthesia, which can be useful in some specific population (e.g., children, obese, allergics, etc.) [72].

Furthermore, the molecular biomarker analysis of biopsy tissue samples allows for the profiling of cytokines and other proteins linked with Th2 immune response in EoE, like IL-13 and eotaxin-3 [73]. Such analyses improve diagnostic specificity, identify disease activity levels, and may assist in predicting treatment responses [74].

Lastly, immunohistochemical staining techniques allow for a more detailed analysis of immune cell infiltration, especially eosinophils and mast cells, in esophageal biopsies [75]. These techniques provide diagnostic sensitivity, enabling a more precise differentiation between EoE and other inflammatory esophageal conditions [67,73,75].

In summary, these adjunctive tools may significantly expand the diagnostic landscape for EoE, improving accuracy, disease monitoring, and enabling a more individualized approach to patient management.

## 4. Endoscopic Management of Eosinophilic Esophagitis

### 4.1. Endoscopic Dilation

Esophageal dilation is a critical therapeutic approach for managing fibrostenotic complications in EoE, targeting symptoms such as dysphagia and food impaction. This procedure aims to mechanically increase the diameter of the esophageal lumen and improve the swallowing function and overall quality of life of affected patients [30]. Dilation is indicated for patients suffering from significant esophageal narrowing, strictures, or a reduced esophageal caliber due to chronic inflammation and fibrotic remodeling [76]. This intervention is especially valuable in patients who do not achieve sufficient symptom relief with anti-inflammatory treatments [33].

**Figure 1:**
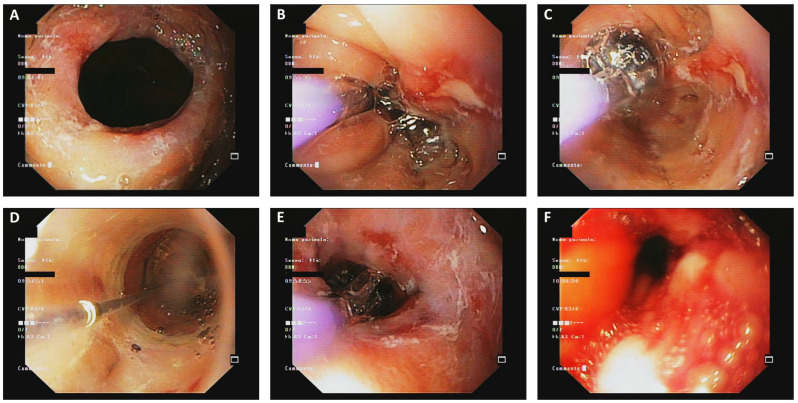
Endoscopic balloon dilation using a through-the-scope (TTS) balloon for an esophageal stricture. (**A**) Initial endoscopic view of the esophageal stricture, showing a concentric narrowing with edematous mucosa. (**B**,**C**) Introduction and progressive inflation of the dilation balloon through the working channel of the endoscope. Balloon expansion leads to the widening of the stenotic lumen with visible superficial mucosal tears. (**D**) Endoscopic monitoring during dilation, with the balloon maintained in position for a predetermined time. (**E**) Post-dilation appearance, showing an improved luminal caliber with areas of mucosal disruption. (**F**) Immediate follow-up view, revealing mild bleeding and inflamed tissue following the procedure.

The procedure can be performed using a variety of tools, including Savary–Guillard or Maloney bougies and through-the-scope (TTS) balloons [77]. Each method has specific advantages, as bougies provide tactile feedback about the degree and location of esophageal narrowing, while TTS balloons (Figure 1) allow for controlled radial dilation and immediate visual assessment of mucosal tearing [78,79].

**Figure 2:**
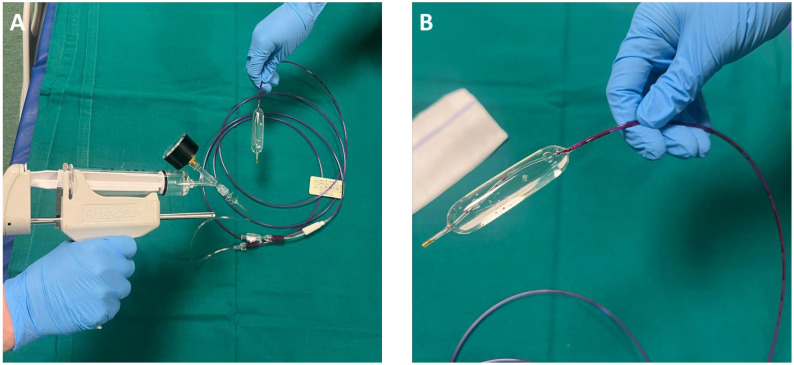
Through-the-scope (TTS) balloon dilation system preparation. (**A**) Setup of the TTS balloon dilation system, showing the inflation device connected to the balloon catheter. The system allows controlled inflation by adjusting pressure via a manometer. (**B**) Close-up view of the TTS balloon in its fully inflated state, demonstrating its cylindrical shape, which facilitates the uniform expansion of the stenotic segment.

The choice of tool often depends on the operator expertise and the specific characteristics of the esophageal narrowing. An important meta-analysis included 37 articles covering 2034 esophageal dilations in 977 patients with EoE, comparing the safety of bougie and balloon dilation. The overall perforation rate was low (0.033%), with no significant difference between bougie (0.022%) and balloon dilation (0.059%). No perforation required surgical intervention, and post-procedural hospitalization rates were comparable [80]. Nevertheless, bougies may be more practical to use in patients with severe and long strictures, while pneumatic balloons (Figure 2) may be more viable for short strictures [63,81].

Overall, dilation should be performed gradually, often over multiple sessions, to reduce the risk of complications. Sessions are typically spaced three to four weeks apart, and the goal is to achieve an esophageal diameter of 16–18 mm, which is sufficient to permit a regular bolus progression and alleviate the fear of food impaction [31]. The appearance of mucosal tears—once considered complications—is now recognized as a marker of effective dilation. Nevertheless, the procedure should be stopped if significant resistance or bleeding occurs [82].

Patients should be thoroughly evaluated before dilation, with endoscopy used to identify the location and severity of strictures. Additional imaging, including barium esophagogram, can be valuable in cases where endoscopic visualization is insufficient [83]. A systematic review and meta-analysis highlighted that the procedure is highly effective, with 95% of patients experiencing clinical improvement after dilation. This investigation included a total of 845 EoE patients, including 87 pediatric cases, underwent 1820 esophageal dilations. The median number of procedures per patient was 3, ranging from 1 to 35. One of the most significant aspects of this study is the safety profile of endoscopic dilation. Although past reports raised concerns about complications, the data from this meta-analysis demonstrate that serious adverse events are very rare. The rate of perforation was 0.38%, hemorrhage occurred in only 0.05% of cases, and hospitalization was required in 0.67% of patients. Importantly, no deaths were reported. The study also highlighted that chest pain occurred in about 9.3% of cases, but only a very small percentage required hospitalization for pain management [84].

Despite its efficacy in addressing the mechanical effects of esophageal remodeling, such as reduced compliance and luminal narrowing, dilation does not treat the underlying eosinophilic inflammation. To note, evidence suggests that the long-term success of dilation depends on its integration into a broader treatment strategy that addresses both the inflammatory and structural aspects of EoE. Studies report that dilation alone provides symptom relief for an average of two years, although concurrent anti-inflammatory therapy significantly enhances outcomes and reduces the risk of recurrent strictures [44]. Notably, in cases with severe fibrostenotic disease, early and repeated dilation can restore esophageal diameter, allowing a better tolerability of medical or dietary treatments [85].

Current guidelines recommend esophageal dilation for patients with strictures who experience persistent dysphagia or food impactions despite medical or dietary therapies [31,44,86]. Finally, we should highlight that considerable variability in clinical practice remains regarding dilation strategies, timing, and its integration with anti-inflammatory therapies, reflecting gaps in current guidelines and necessitating further clinical trials. Emerging techniques, such as EndoFlip™, may further enhance the selection of candidates for dilation by providing objective assessments of esophageal distensibility and compliance [66].

### 4.2. Monitoring Treatment Response

Endoscopy plays a fundamental role in monitoring medical and endoscopic treatment response in patients with EoE. Given the chronic and progressive nature of the disease, regular endoscopic evaluations are essential to assess mucosal healing, detect fibrosis-related complications, and guide further therapeutic decisions.

The 1st EoETALY Consensus on the Diagnosis and Management of Eosinophilic Esophagitis suggests a planned approach to endoscopic monitoring based on treatment phase and disease status. After 12 weeks of medical therapy initiation, a follow-up endoscopy is recommended to evaluate histological remission and treatment adjustments should be considered whether eosinophil counts remain elevated (>15 eosinophils per high-power field). For patients in remission, long-term monitoring through endoscopic surveillance is advised every 12–24 months, particularly for those with a history of fibrosis, strictures, or recurrent symptoms. If treatment is modified, another endoscopic assessment at 12 weeks can help evaluate response. More frequent endoscopies may be necessary for patients with persistent symptoms or refractory disease [31]. On the other hand, after endoscopic therapy, a follow-up endoscopy is recommended to assess treatment response and identify any late complications, with further surveillance frequency tailored to the patient clinical and histological status [27].

Notably, several reports have highlighted the only modest accuracy of symptoms in predicting histological remission in EoE, potentially owing to confounding elements such as anxiety linked to specific symptoms and esophageal dysmotility [63]. Therefore, the main treatment goals should consider symptoms associated with a reduction in eosinophil density below 15 eosinophils per high-power field [86]. While this threshold is commonly used for diagnosis EoE remission, recent findings suggest that even lower levels may correlate with persistent disease activity [87]. In an effort to enhance the histological evaluation beyond peak eosinophil counts, the EoE histology scoring system (EoEHSS) has been developed [88]. This scoring system underscores the significance of a comprehensive histological analysis, given that specific microscopic features—such as basal cell hyperplasia—can be linked to ongoing symptoms even when eosinophil counts are relatively low [89]. Moreover, the EoEHSS exhibits excellent interobserver agreement, discerns treated from untreated EoE more effectively than peak eosinophil counts, and correlates with EoE symptom indices [90]. Consequently, prioritizing detailed histological examination is pivotal for guiding therapy modifications and achieving both minimal eosinophil presence and improvement in additional histologic parameters.

One recognized drawback of relying on esophageal biopsies is that they often contain limited amounts of submucosal tissue (including the lamina propria and muscularis), which can hinder the full appraisal of the esophageal wall [29,43]. To address this limitation, EndoFlip™ has emerged as a valuable tool. Actually, incorporating EndoFlip™ into patient evaluations allows for the objective assessment of morphological changes in the esophagus before and after therapy, complementing both symptomatic and histological measures of treatment response [91]. We feel that, with the rising prevalence of EoE and the corresponding increase in endoscopic procedures, the optimal biopsy strategy for follow-up patients remains a subject of ongoing debate. While clear guidelines exist for initial diagnostic biopsies, follow-up practices aimed at evaluating disease activity and therapeutic response vary considerably across clinical centers. This heterogeneity reflects both the patchy nature of eosinophilic infiltration throughout the esophagus and the lack of consensus regarding the minimum number and ideal locations of biopsies required to ensure a reliable assessment during follow-up.

### 4.3. Next-Generation Endoscopic Therapies

Recently, a new hydrostatic balloon dilator has been used to treat esophageal narrowing and strictures. The EsoFlip^®^ (Medtronic Inc, Shoreview, MN, USA) combines the impedance planimetry technology of the EndoFlip™ with a stiffer balloon to generate higher but controlled endoluminal pressure. This device presents several advantages over traditional TTS balloons or Savary bougies: (a) objective measurement of the diameter of the strictures; (b) objective evaluation of the intraprocedural behavior of the esophageal wall and strictures; (c) objective evaluation of the response to treatment; (d) precise controlled filling of the balloon to the desired diameter, which also means to provide a wide range of dilation diameters with the same catheter; (e) constant control of the correct placement of the balloon on the EndoFlip™ monitor, that is, no need for fluoroscopy; and (f) visualization of the shape of the lumen on the EndoFlip™ monitor [92]. Moreover, likewise with the TTS balloon, it allows the direct visualization of the lumen/stenosis through the balloon with the endoscope. At the moment, 10, 20, and 30 mm probes are available in the market. After an initial partial filling volume of 20/30 mL (for the 20 and 30 mm balloon, respectively), the balloon can be filled with an increment of 1 mL at a time or higher according to the target diameter. The full dilation diameter of 20 and 30 mm is reached at 45 and 75 mL volume values, respectively [93].

There are limited data evaluating the clinical outcomes of EsoFlip^®^ for EoE strictures. Previous observational studies performed on patients with achalasia and EGJOO showed a pooled clinical success rate of about 69% with a good safety profile [93]. A recent retrospective study compared EsoFlip^®^ dilation with TTS balloon dilation combined with EndoFlip™ measurement in children affected by esophageal strictures (both congenital and acquired—including eosinophilic esophagitis). EsoFlip^®^ dilation was faster and potentially cheaper than the TTS balloon + EndoFlip™ treatment, requiring less fluoroscopy [94]. It must be noted that EsoFlip^®^ provides immediate volume/diameter measurements, but it needs to be connected to an optional external device for real-time intraluminal pressure measurements. Further randomized controlled trials are required to evaluate EsoFlip^®^ clinical efficacy in EoE patients.

## 5. Patient Experience in Endoscopic Approaches for EoE: Sedation, Tolerance, and Preferences

In EoE, where disease activity often diverges from symptoms, endoscopy remains indispensable, yet the way in which each approach impacts recovery time, safety, and personal preference is increasingly shaping clinical choices. Traditional sedated EGD provides excellent visualization and nearly universal intraprocedural comfort, but patients exchange those advantages for several hours of lost time: pre-procedure fasting, intravenous cannulation, transfer to a recovery area, and the lingering psychomotor effects of anesthetic drugs that preclude driving or working for the remainder of the day [95]. Repetition magnifies inconvenience and heightens cumulative anesthesia exposure, a particular concern in pediatrics where even small risks of neurocognitive sequelae drive family anxiety [96]. By contrast, unsedated transnasal endoscopy compresses the entire visit—topical nasal anesthesia, scope passage, biopsy collection, and brief observation—into roughly one hour, after which adults can return to work and children to school or sports without dietary restrictions or escort requirements [97,98]. Evidence reports no serious adverse events and only mild, self-limited nasal irritation or sore throat, eliminating the respiratory and cardiovascular hazards intrinsic to systemic sedation [72]; we feel that, for parents, the mere avoidance of a recovery bay and pulse-oximeter alarms is a potent source of reassurance. This dramatic reduction in downtime consistently translates into higher satisfaction scores: in cohorts who have experienced both modalities, two-thirds of patients and as many as four-fifths of parents of children with EoE state they would choose transnasal endoscopy for subsequent surveillance [72,98]. Nevertheless, decision-making remains nuanced, as some patients find the idea of being awake during the endoscopy intolerable or suffer from nasal septal deviation or severe gag reflex that precludes successful transnasal endoscopy [72,99].

Finally, emerging non-endoscopic sampling tools, such as the Cytosponge or string tests, promise to push recovery time toward zero and minimize risk further, but until their diagnostic breadth equals that of endoscopy, transnasal endoscopy may represent the optimal balance between information yield and patient-centered experience [100].

We think the ultimate goal is not merely to diagnose and monitor disease but to consider also patients’ personal comfort and practical needs—highlighting the essential role of personalized medicine in enhancing adherence, satisfaction, and quality of life.

## 6. Pediatric Population

While endoscopic management is pivotal for diagnosis and management in EoE, pediatric and adult approaches differ in aspects such as sedation methods, endoscope equipment, biopsy techniques, and therapeutic interventions.

Children generally require deep sedation or general anesthesia for upper endoscopy, to ensure patient comfort and safety, while adults typically undergo endoscopy with moderate conscious sedation [101]. Given concerns about repeated anesthesia exposure in early childhood [102], pediatric centers are exploring less invasive options like unsedated transnasal endoscopy for follow-up assessments [102].

Moreover, pediatric endoscopes have a smaller diameter and working channel than adult endoscopes [103]. This necessitates specialized pediatric instruments (e.g., smaller biopsy forceps), but, otherwise, the endoscopic technique (esophageal intubation and inspection) is similar [104]. Procedures in young children are often performed in an operating room with pediatric anesthesiology support, whereas adult endoscopies occur in endoscopy units.

The diagnostic protocol is similar as multiple biopsies from at least two esophageal regions (usually at least six in total) are recommended in both children and adults to maximize diagnostic yield [102]. Histologic criteria for EoE (≥15 eosinophils per high-power field) are the same across age groups. One distinction is that children with EoE often have more inflammatory endoscopic features (edema, furrows, exudates) and fewer strictures initially, while adults, who often have longer disease duration, more frequently display rings and strictures from fibrostenotic remodeling [105].

Form a therapeutic point of view, dilation was historically approached cautiously due to perforation concerns in pediatric patients with EoE, but recent studies show it can be performed safely in children [102]. A large pediatric series reported no perforations and only mild, transient adverse events during 68 dilation procedures in EoE patients [106]. Thus, with a careful stepwise technique, children can benefit from dilation similarly to adults. In pediatric EoE, therapeutic endoscopic procedures (dilation or food bolus removal) are performed under general anesthesia with endotracheal intubation for airway protection [102], whereas adults often tolerate these interventions with deep sedation alone. Aside from anesthesia, the technical approach to stricture dilation and impaction management is similar in both populations. Overall, the major procedural differences reflect the need for pediatric-specific equipment and sedation, while the therapeutic goals are the same.

## 7. Future Directions and Conclusions

The field of endoscopic management in EoE has evolved significantly, driven by advances in diagnostic techniques, therapeutic interventions, and personalized treatment strategies. However, several challenges remain, necessitating further research and innovation to optimize patient outcomes [44].

One of the key areas for future exploration is the refinement of non-invasive diagnostic and monitoring tools. Future studies, possibly prospective, should focus on validating these innovative methods in larger cohorts and integrating them into clinical practice to improve long-term disease monitoring while minimizing patient burden [107]. Another crucial avenue is the integration of functional assessments into routine practice. Tools such as EndoFlip™ provide valuable insights into esophageal distensibility, correlating structural changes with symptomatic and histologic disease activity. Further research should establish standardized EndoFlip™, cut-off values for guiding treatment escalation and determining the need for dilation therapy [108].

Other emerging diagnostic tools complement these approaches. Confocal laser endomicroscopy enables the real-time, in vivo imaging of esophageal mucosa at a cellular level, potentially reducing the need for invasive biopsies [109]. Similarly, capsule endoscopy offers a non-invasive method for visualizing mucosal abnormalities in the esophagus, though its application for EoE is still under investigation [107]. Liquid biopsy techniques, which analyze blood or saliva samples for eosinophil-derived proteins or genetic markers, present an exciting avenue for completely non-invasive diagnostics and disease monitoring [110].

Additionally, artificial intelligence (AI) is gaining traction in endoscopic diagnostics. AI-powered systems can analyze endoscopic images with enhanced precision, identifying subtle mucosal changes indicative of EoE [42,111,112]. Endoscopic ultrasound (EUS) offers another advanced diagnostic modality, providing insights into esophageal wall remodeling and fibrosis, particularly in fibrostenotic disease [26].

Advances in genomic and proteomic profiling, as well as microbiome analysis, represent promising areas of research. These methods aim to uncover new biomarkers and stratify patients based on genetic predispositions or immune responses, potentially leading to personalized treatment strategies [113,114].

In terms of therapeutic strategies, endoscopic dilation remains an essential option for fibrostenotic disease, but its role alongside emerging biologic therapies requires further clarification. As monoclonal antibodies such as dupilumab, cendakimab, and benralizumab continue to demonstrate efficacy in reducing eosinophilic inflammation, future studies should investigate whether early biologic intervention can prevent or delay fibrostenotic complications, potentially reducing the need for serial dilations [44]. Moreover, the use of novel endoscopic approaches, such as EsoFlip^®^-assisted dilation, may provide more precise and controlled esophageal remodeling, warranting comparative studies against conventional bougie or balloon dilation techniques [92].

Lastly, a multidisciplinary approach incorporating gastroenterologists, allergists, dietitians, psychologists, and patient-reported outcomes is considered pivotal in advancing the field. Standardized treatment algorithms incorporating dietary, pharmacologic, and endoscopic interventions tailored to individual patient phenotypes are needed. Collaborative efforts to advance histologic remission criteria beyond peak eosinophil counts and incorporate broader markers of tissue remodeling and inflammation are warranted [115].

In conclusion, while significant progress has been made in the endoscopic management of EoE, ongoing research is fundamental to refine diagnostic precision, optimize therapeutic interventions, and improve patient-centered outcomes. The integration of novel technologies, personalized treatment strategies, and multidisciplinary care will shape the future landscape of EoE management, ultimately enhancing long-term disease control and quality of life for affected individuals.
